# Supplementary material for: Dynamic control of IDP interaction network via diverse binding pathways
Source: Nat Commun. 2026 Feb 26;17:3249. doi: 10.1038/s41467-026-69946-4 (PMC13062061; doi:10.1038/s41467-026-69946-4)
Supplement: Supplementary file 1 — Supplementary Information [file 41467_2026_69946_MOESM1_ESM.pdf]

Supplementary Materials for

Dynamic control of IDP interaction network via diverse binding pathways

Jae-Yeol Kim and Hoi Sung Chung [*].

*Corresponding author: chunghoi@niddk.nih.gov

The PDF file includes:

Supplementary Figure 1 to 7
Supplementary Table 1 to 4

a

AviTag — Linker

GHMGMSGLNDIFEAQKIEWHE SSGLVAGGGGSGGGGSGGGGS

UPLSQETFSDLWKLLPENNVLSPLPSQAMDDLMLSPDDIEQWFTEDPGPDC

TAD

b

GSUSPQESRRLSIQRAIQSLVHAAQCRNANCSLPSCQKMKRVVQHTKGCKRKTNGGCPVC

KQLIALAAYHAKHCQENKCPVPFCLNIKHKLRQQQ

Taz2

c

GSQIPASEQETLVRPKPLLLKLLKSVGAQKDTYTMKEVLFYLGQYIMTKRLYDEKQQHIV

YCSNDLLGDLFGVPSFSVKEHRKIYTMIYRNLVVVNQQESSDSGTSVSEN

Mdm2

d

AviTag — Linker

GHMGMSGLNDIFEAQKIEWHE SSGLVAGGGGSGGGGSGGGGS

QAMDDLMLSPDDIEQWFTEDPGPDC

AD2

Supplementary Figure 1. Amino acid sequences of proteins. **a**, TAD sequence. An unnatural amino acid (green U), 4-acetylphenylalanine, and a cysteine residue (orange C) are appended to the N- and C-termini of TAD. Biotin is attached to the lysine residue (blue K) in the AviTag sequence, which is separated from TAD sequence by a flexible linker, providing space between the surface and the immobilized proteins and minimizing immobilization effect on binding. U and C were labeled with the donor (Alexa 488) and acceptor 1 (Alexa 594), respectively. The residues inside the orange and light green rectangles form α helices upon binding to Taz2 and Mdm2, respectively. **b**, Taz2 sequence. Acceptor 2 (CF680R) was attached to 4-acetylphenylalanine residue (red U). **c**, Mdm2 sequence. **d**, The sequence of AD2 domain of TAD. Alexa 488 was attached to the C-terminal cysteine residue (green C). The residues inside the orange rectangle form α helix upon binding to Taz2.

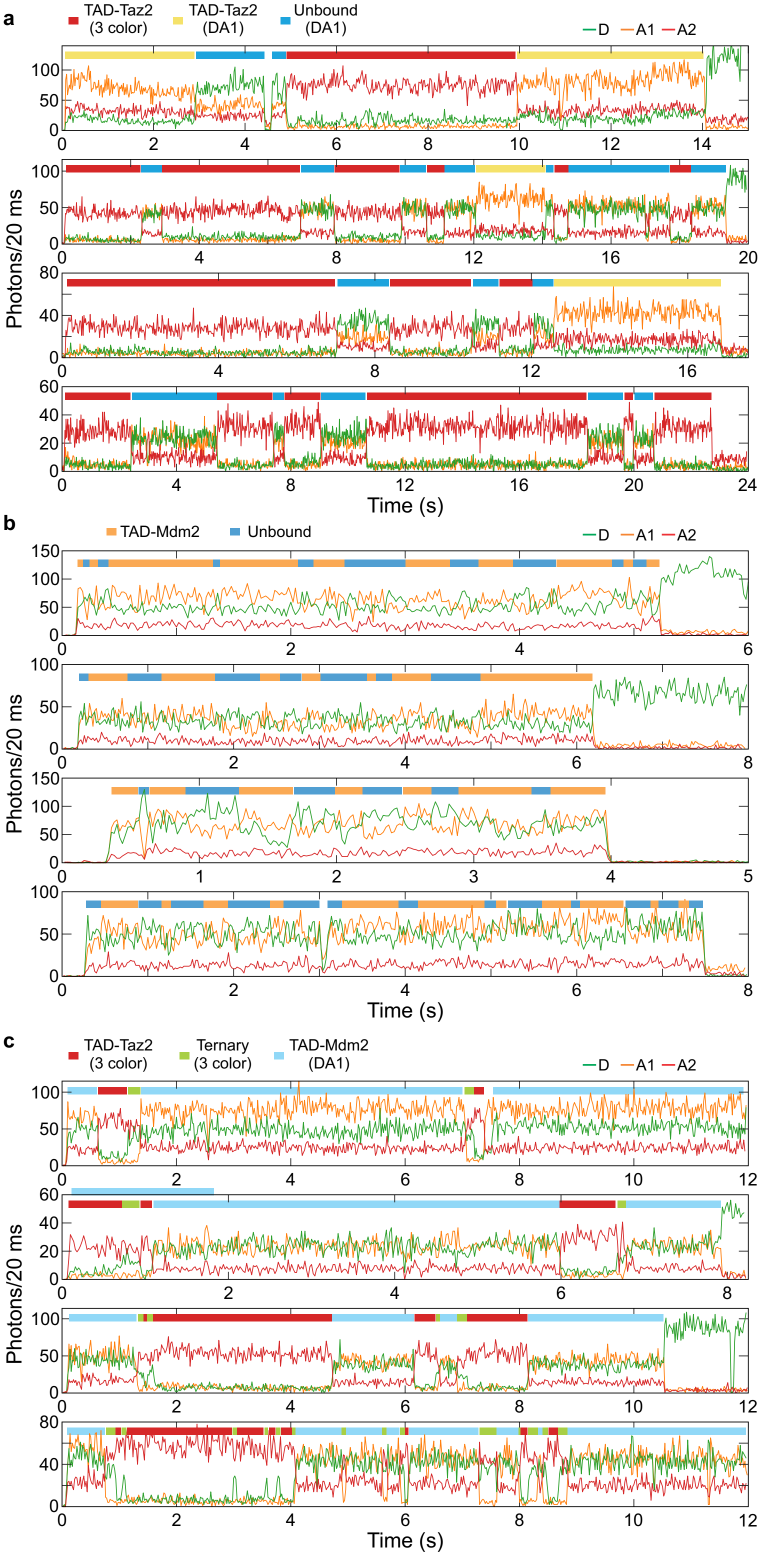

Supplementary Figure 2. Additional binned (20 ms bin time) fluorescence trajectories of TAD, Taz2, and Mdm2 binding. a, Binding of TAD and Taz2. [Taz2] = 17 nM. **b**, Binding of TAD and Mdm2. [Mdm2] = 200 nM. **c**, Ternary complex formation. [Taz2] = 17 nM and [Mdm2] = 2 μM. See Fig. 2 legend for additional details.

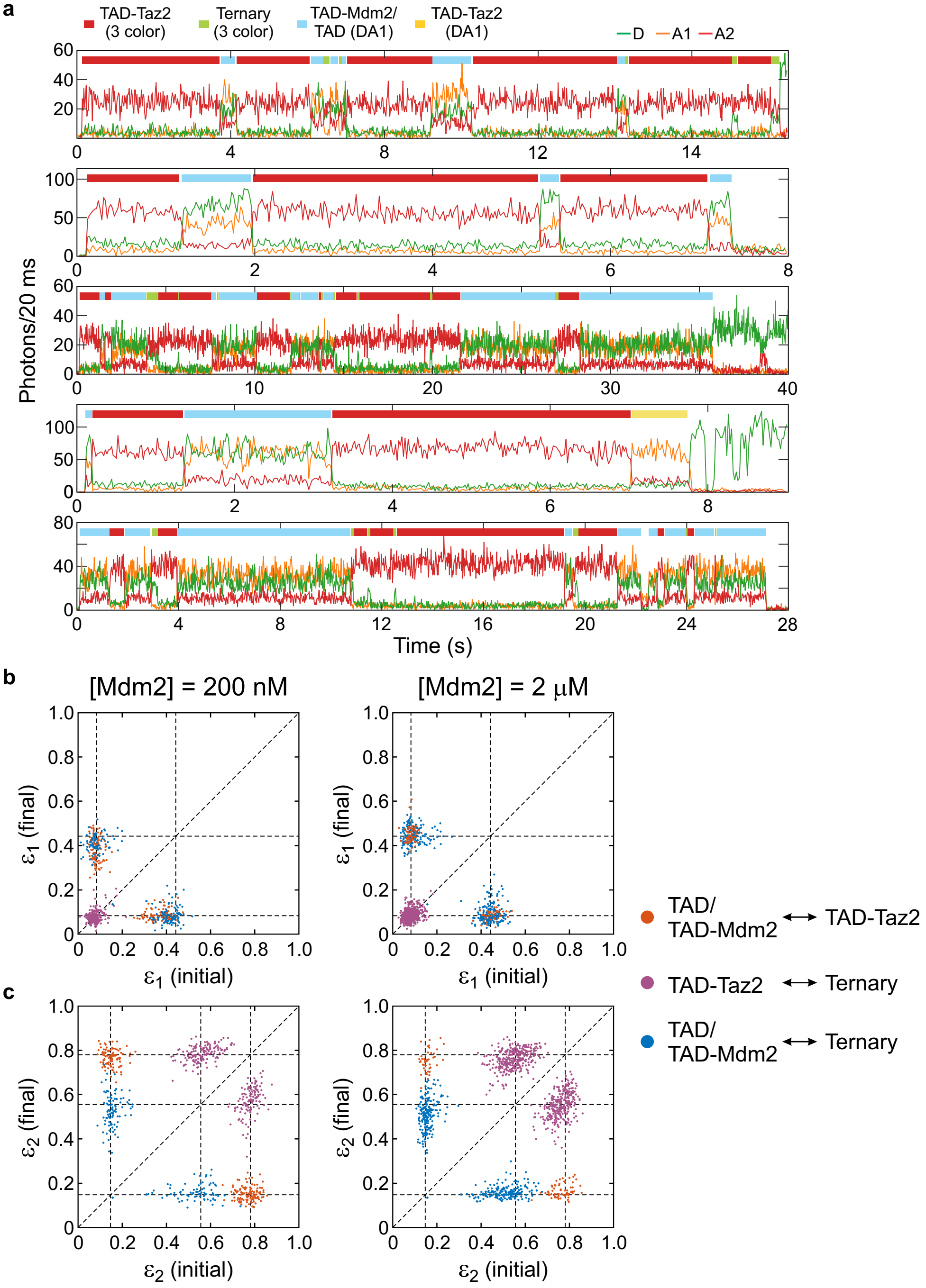

Supplementary Figure 3. Ternary complex formation at [Mdm2] = 200 nM. a, Binned (20 ms bin time) fluorescence trajectories. [Taz2] = 17 nM and [Mdm2] = 200 nM. 110 trajectories were analyzed. See Fig. 2 legend for additional details. **b, c,** Two-dimensional transition map of (**b**) ε_1 and (**c**) ε_2 at [Mdm2] = 200 nM (left) and 2 μM (right). The distributions are similar at both concentrations except for the broader and lower ε_1 distribution of red and blue dots at 200 nM, indicating the increased contribution by the free TAD compared to that at 2 μM. More transitions along the competitive pathway (red dots) and less transitions along the allosteric pathways (blue and purple dots) are observed at [Mdm2] = 200 nM than at 2 μM. Source data for (**b**) and (**c**) are provided as a Source Data file.

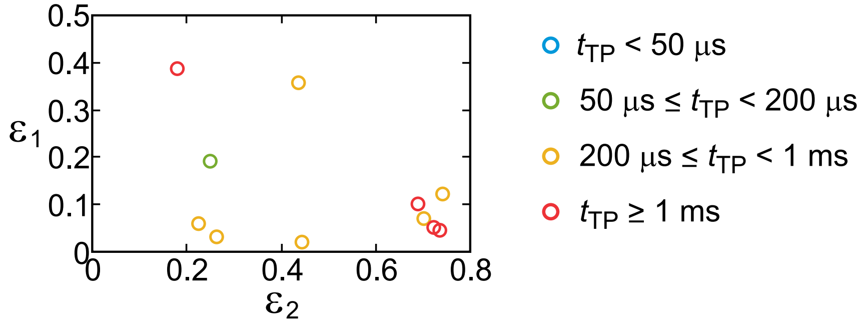

Supplementary Figure 4. Measurement of TPs of TAD-Taz2 binding at [NaCl] = 15 mM. The distribution of ε_1 and ε_2 values of individual TPs of TAD and Taz2 binding measured at [NaCl] = 15 mM. The color of each data point indicates the range of t_{TP}. 19 transitions were analyzed. Only the data with $\Delta \ln L > 0.5$ are included in the plot. Source data are provided as a Source Data file.

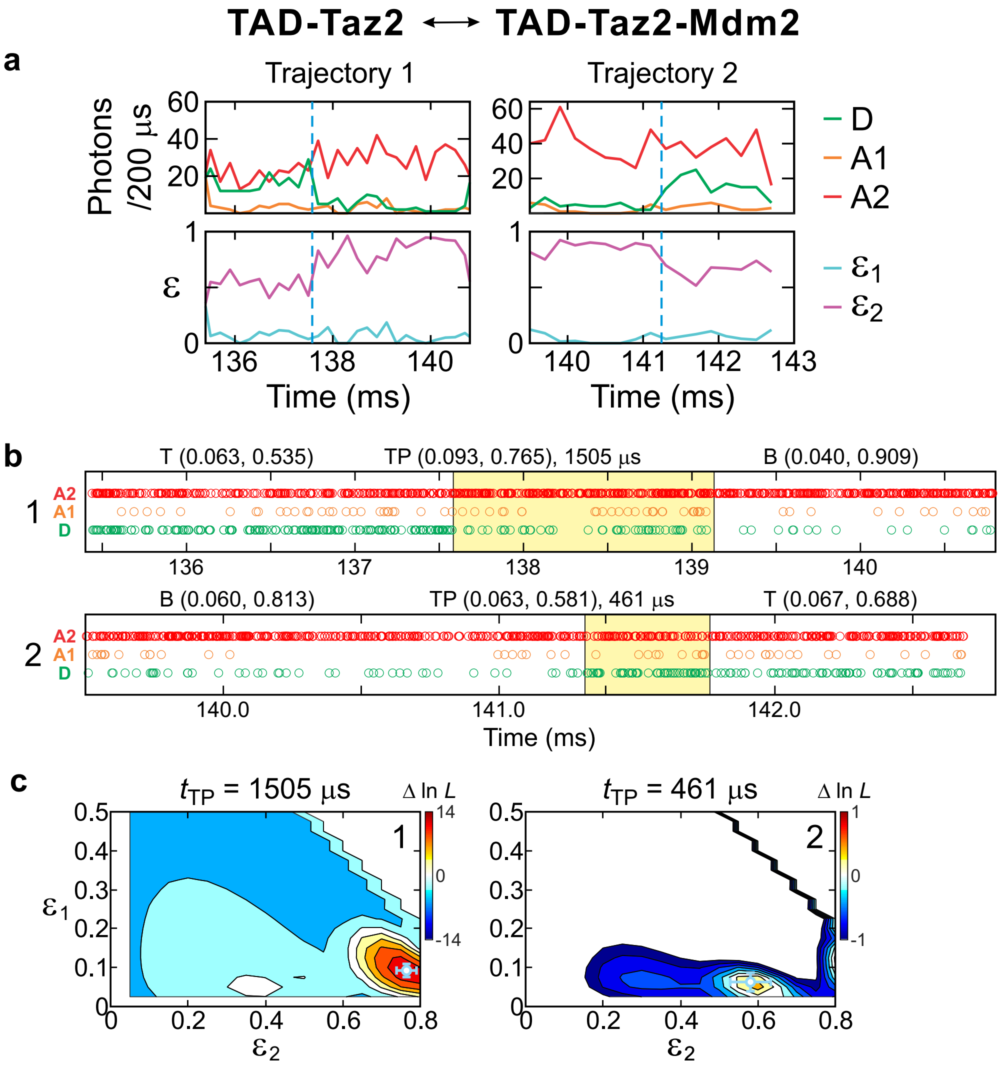

Supplementary Figure 5. Measurement of TPs between TAD-Taz2 and ternary complexes. a, Representative binned (200 µs bin time) fluorescence, ε_1, and ε_2 trajectories of high illumination intensity three-color FRET experiments. **b**, Photon trajectories near the transitions of the trajectories in (**a**). Photon trajectories are separated into the unbound state, TP (yellow shaded region), and the bound state using the t_{TP} and acceptor fraction values determined from the maximum likelihood analysis (see Methods). The optimized acceptor fractions (ε_1, ε_2) of the three states and t_{TP} are listed above each trajectory. **c**, The 2D likelihood plot as functions of ε_1 and ε_2 at the optimized t_{TP} for the three transitions in (**b**). $\Delta \ln L$ (= $\ln L(\tau_S)$ – $L(0)$) is the difference of the log likelihood between the one-intermediate model and the instantaneous transition model (t_{TP} = 0). The log likelihood difference in the white area outside the likelihood peaks is smaller than the minimum value of the color bar. Error bars indicate SDs obtained from the curvature at the maximum of the likelihood function.

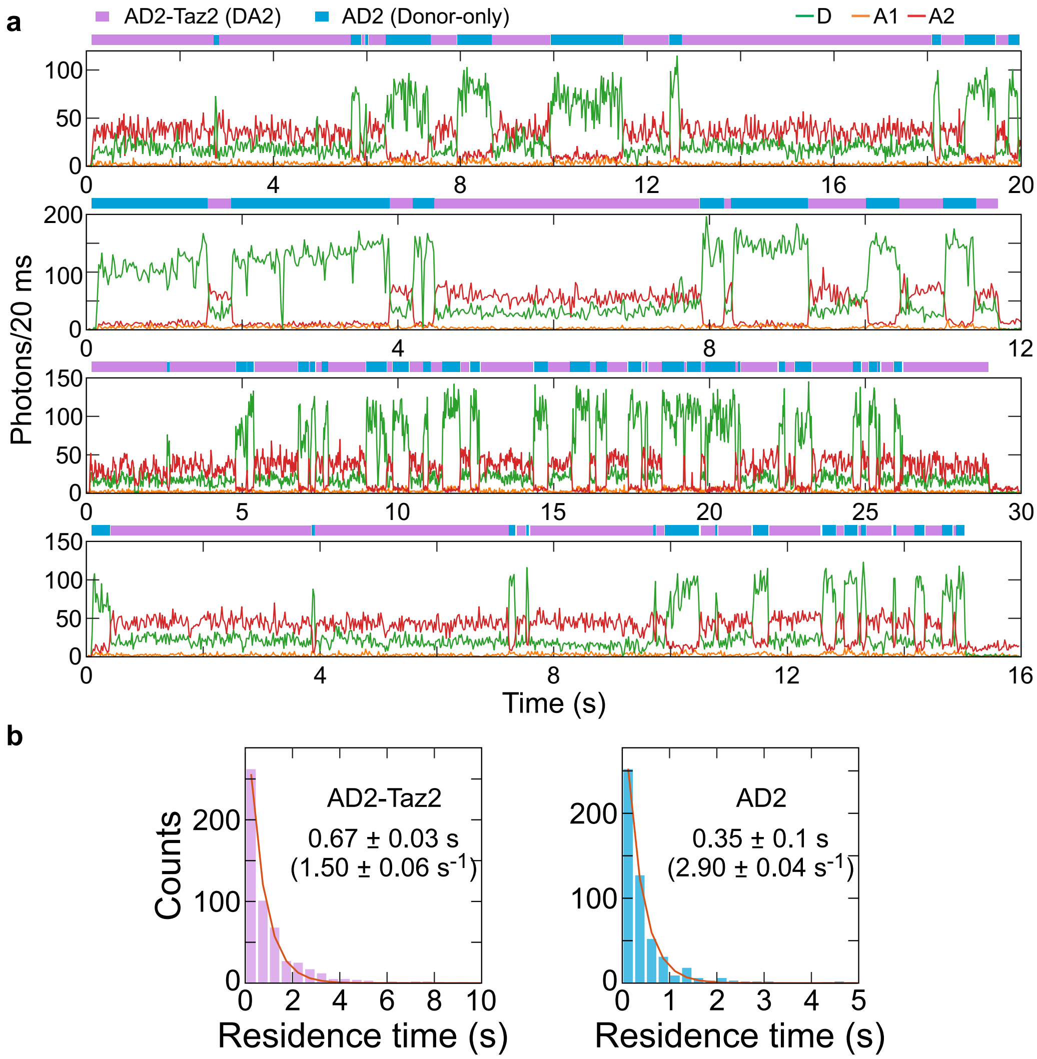

Supplementary Figure 6. AD2-Taz2 binding experiment. a, Representative binned (20 ms bin time) fluorescence trajectories of two-color FRET experiments of AD2-Taz2 binding. [Taz2] = 60 nM. [NaCl] = 15 mM. 125 trajectories were analyzed. Donor, A1, and A2 fluorescence signals are shown in green, orange, and red, respectively. Segments of the AD2-Taz2 bound state (DA2 and the unbound state (Donor-only) are indicated by purple and light blue bars above the trajectories, respectively. **b**, The waiting time distributions in the AD2-Taz2 bound state and unbound state. Red lines indicate exponential fitting. Errors are standard deviations obtained from fitting.

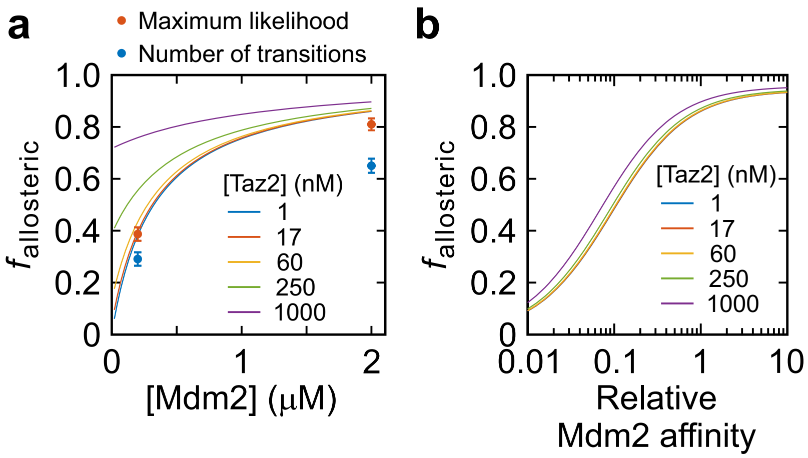

Supplementary Figure 7. Relative flux of Taz2 and Mdm2 exchange along the allosteric pathway. a, Dependence on the Mdm2 concentration. Blue and red dots are the experimental values obtained from the number of transitions assigned in binned trajectories and from the maximum likelihood analysis of photon trajectories, respectively (see *Calculation of the relative flux involving the ternary complex* in Methods). The total transitions were 306 at both Mdm2 concentrations and the transitions along the allosteric pathways were 89 and 199 at 200 nM and 2 μM, respectively. Errors are the binomial errors (blue) and the standard deviations obtained from the diagonal elements of the covariance matrix calculated at the maximum of the likelihood function (red). **b**, Dependence on the relative Mdm2 affinity with respect to that in this work (K_D = 186 nM) at [Mdm2] = 2 μM. Source data are provided as a Source Data file.

Supplementary Table 1. Comparison of dissociation constants (K_d, nM) of the TAD-Taz2 and TAD-Mdm2 complexes and experimental conditions

TAD residues	Taz2	Mdm2	Condition	Study
13 - 61	10.9 (± 0.8)	186 (± 5)	20 mM Tris-HCl, pH 7, 15 mM NaCl, 22 °C	This work
13 - 61	26 (± 7)	230 (± 20)	20 mM Tris-HCl, pH 8.0, 50 mM NaCl, 35 °C	Ferreon *et al.* [Ref. 13]
1 - 57	27 (± 10)	160 (± 40)	50 mM MES, pH 6.8, 100 mM NaCl, 23 °C	Teufel *et al.* [Ref. 12]
13 - 61	20 (± 1.6)	–	–	Lee *et al.* [Ref. 19]
1 - 39	–	70 (± 20)	25 mM MES, pH 6.8, 100 mM NaCl, 4 °C	Sakaguchi *et al.* [Ref. 18]
15 - 29	–	575 (± 19)	10 mM sodium phosphate, pH 7.5, 150 mM NaCl, 15 °C, ITC	Schon *et al.* [Ref. 17]
15 - 29	–	220 (± 8)	10 mM sodium phosphate, pH 7.5, 150 mM NaCl, 15 °C, Stopped-flow kinetics	Schon *et al.* [Ref. 17]

Supplementary Table 2. Rate constants determined from TAD-Taz2 binding experiment and ternary complex formation experiment.

Rate constants‡	Experiment				
	TAD-Taz2	TAD-Mdm2	Ternary		AD2-Taz2
			200 nM†	2 μM†	
$k_{\mathrm{a,Taz2}}^{\mathrm{TAD\text{-}Taz2}}$ ($\times 10^7$ $M^{-1}s^{-1}$)	3.46 (± 0.14)				
$k_{\mathrm{d,Taz2}}^{\mathrm{TAD\text{-}Taz2}}$ (s^{-1})	0.38 (± 0.02)		0.134 (± 0.005)	0.222* (± 0.003)	
$k_{\mathrm{a,Mdm2}}^{\mathrm{TAD\text{-}Mdm2}}$ ($\times 10^7$ $M^{-1}s^{-1}$)		4.2 (± 0.2)			
$k_{\mathrm{d,Mdm2}}^{\mathrm{TAD\text{-}Mdm2}}$ (s^{-1})		7.9 (± 0.3)			
$k_{\mathrm{a,Taz2}}^{\mathrm{Ternary}}$ ($\times 10^7$ $M^{-1}s^{-1}$)			5.21 (± 0.25)	3.24 (± 0.19)	
$k_{\mathrm{d,Taz2}}^{\mathrm{Ternary}}$ (s^{-1})			2.65 (± 0.07)	2.24 (± 0.04)	
$k_{\mathrm{a,Mdm2}}^{\mathrm{Ternary}}$ ($\times 10^7$ $M^{-1}s^{-1}$)			0.155 (± 0.006)	0.108 (± 0.002)	
$k_{\mathrm{d,Mdm2}}^{\mathrm{Ternary}}$ (s^{-1})			4.16 (± 0.10)	3.61 (± 0.07)	
$k_{\mathrm{a,Taz2}}^{\mathrm{AD2\text{-}Taz2}}$ ($\times 10^7$ $M^{-1}s^{-1}$)					4.83 (± 0.07)
$k_{\mathrm{d,Taz2}}^{\mathrm{AD2\text{-}Taz2}}$ (s^{-1})					1.50 (± 0.06)

Errors are standard deviations calculated using fitting errors and number of transitions when transitions occur from one state to two different states (binomial distribution).

‡ For a reaction, $\mathrm{X} + \mathrm{L} \rightleftarrows \mathrm{C}$, the association and dissociation rate constants are expressed as $k_{\mathrm{a,L}}^{\mathrm{C}}$ and $k_{\mathrm{d,L}}^{\mathrm{C}}$. C represents the bound complex: TAD-Taz2, TAD-Mdm2, and Ternary. L represents the binding partner that binds to form a complex C or dissociates from a complex C.

* Adjusted to 0.136 to satisfy the detailed balance in the kinetic scheme in Fig. 2i (see Methods) for the calculation of the relative flux in Supplementary Fig. 7.

† Mdm2 concentration

Supplementary Table 3. Maximum likelihood parameters for the determination of the fraction of transitions along the allosteric pathway in the ternary complex formation using the transition path analysis.

[Mdm2]	200 nM	2 μM
ε_{1B}	0.082 (± 0.001)	0.082 (± 0.001)
ε_{1U}	0.435 (± 0.001)	0.467 (± 0.001)
ε_{2B}	0.791 (± 0.001)	0.785 (± 0.001)
ε_{2U}	0.163 (± 0.001)	0.165 (± 0.001)
$t_{allosteric}$ (ms)	86.3 (± 7.0)	92.1 (± 5.3)
$t_{competitive}$ (ms)	11.8	11.8
$\varepsilon_1^{allosteric}$	0.068 (± 0.001)	0.073 (± 0.001)
$\varepsilon_1^{competitive}$	0.316 (± 0.002)	0.365 (± 0.007)
$\varepsilon_2^{allosteric}$	0.593 (± 0.003)	0.561 (± 0.002)
$\varepsilon_2^{competitive}$	0.114 (± 0.002)	0.116 (± 0.004)
$f_{allosteric}$	0.387 (± 0.026)	0.810 (± 0.023)

Errors are standard deviations obtained from the diagonal elements of the covariance matrix calculated at the maximum of the likelihood function.

Supplementary Table 4. Maximum likelihood parameters for the determination of the transition path times and acceptor fractions of TAD-Taz2 binding and ternary complex formation.

	TAD ↔ TAD-Taz2	TAD ↔ TAD-Taz2	TAD-Taz2 ↔ TAD-Taz2-Mdm2	
[NaCl] (mM)	15	50	15	
	Double TP	Double TP	Single TP	Double TP
ε_{1B}	0.071 (± 0.003)	0.094 (± 0.002)	0.074 (± 0.002)	0.074 (± 0.002)
ε_{1U}	0.336 (± 0.018)	0.406 (± 0.002)	0.068 (± 0.001)	0.073 (± 0.001)
ε_{2B}	0.846 (± 0.006)	0.817 (± 0.004)	0.822 (± 0.004)	0.819 (± 0.005)
ε_{2U}	0.172 (± 0.009)	0.132 (± 0.001)	0.596 (± 0.003)	0.586 (± 0.004)
k_{b1} (ms^{-1})	56 (± 72)	53 (± 6)	52	52
k_{b2} (ms^{-1})	30 (± 12)	163 (± 29)	83	83
p_{b1}*	0.950 (± 0.017)	0.986 (± 0.001)	0.989 (± 0.002)	0.989 (± 0.002)
p_{b2}*	0.992 (± 0.002)	0.993 (± 0.001)	0.996 (± 0.05)	0.996 (± 0.008)
t_{TP1} (μs)	174 (± 71)	90 (± 19)		800 (± 140)
t_{TP2} (μs)	1510 (± 452)	1510 (± 230)	921 (± 98)	1150 (± 160)
ε_{1TP1}	0.067 (± 0.021)	0.048 (± 0.010)		0.041 (± 0.004)
ε_{1TP2}	0.123 (± 0.009)	0.093 (± 0.003)	0.077 (± 0.003)	0.079 (± 0.003)
ε_{2TP1}	0.240 (± 0.035)	0.567 (± 0.027)		0.651 (± 0.008)
ε_{2TP2}	0.680 (± 0.018)	0.747 (± 0.005)	0.721 (± 0.006)	0.717 (± 0.007)
f_{TP1}	0.368 (± 0.138)	0.431 (± 0.084)		0.251 (± 0.080)

Errors are standard deviations obtained from the diagonal elements of the covariance matrix calculated at the maximum of the likelihood function.

* At a photon count rate of 100 ms^{-1}.
